# Hydrophobic residue substitutions enhance the stability and *in vivo* immunogenicity of respiratory syncytial virus fusion protein

**DOI:** 10.1128/jvi.00087-25

**Published:** 2025-05-28

**Authors:** Qiaoyun Song, Haixia Yang, Haoyue Zhu, Yun Hu, Wenling Shen, Huifeng Cheng, Jialiao Cai, Manlan Qiu, Yueyue Li, Yaolan Li, Wencai Ye, Ying Wang, Wei Tang

**Affiliations:** 1 State Key Laboratory of Bioactive Molecules and Druggability Assessment, Jinan University 47885, Guangzhou, People's Republic of China; 2 Guangdong Province Key Laboratory of Pharmacodynamic Constituents of TCM & New Drugs Research, Guangdong-Hong Kong-Macau Joint Laboratory for Pharmacodynamic Constituents of TCM and New Drugs Research, Jinan University 47885, Guangzhou, People's Republic of China; 3 Center for Bioactive Natural Molecules and Innovative Drugs Research, College of Pharmacy, Jinan University 506479, Guangzhou, People's Republic of China; The Peter Doherty Institute for Infection and Immunity, Melbourne, Australia

**Keywords:** respiratory syncytial virus, viral fusion, prefusion F, vaccine

## Abstract

Respiratory syncytial virus (RSV) entry into host cells is facilitated by viral fusion, wherein the metastable RSV fusion (F) protein undergoes a conformational change from a prefusion state to a highly stable postfusion structure. The prefusion F elicits a more robust human antibody response than its postfusion F and is a primary target for RSV vaccine development. However, the inherent instability of the prefusion F trimer and its low protein expression level in host cells are a significant challenge for developing a high-potency RSV vaccine. Here, we report that the introduction of four hydrophobic residue substitutions in the RSV F protein resulted in a highly stable prefusion F trimer (pre-F-IFLP). This engineered variant exhibits enhanced expression and stability compared to DS-Cav1, with improved thermal stability, increased resistance to acid and base, and extended storage life. Furthermore, pre-F-IFLP induced neutralizing antibody responses 72-fold higher than those elicited by DS-Cav1 following a second booster immunization and fully protected mice against RSV infection.

**IMPORTANCE:**

In this study, we demonstrate that introducing four hydrophobic residue substitutions into the RSV F protein leads to the generation of a highly stable prefusion F trimer (pre-F-IFLP) with improved expression levels in cultured cells and superior stability compared to DS-Cav1, the first-generation prefusion F-stabilized RSV vaccine. Furthermore, pre-F-IFLP induced significantly higher neutralizing antibody responses than DS-Cav1 following both the first and second booster immunizations and conferred complete protection against RSV infection in a mouse model. These findings present an alternative approach for stabilizing the trimeric prefusion F protein, enhancing its expression, and significantly improving its protective efficacy for the prevention of RSV infection *in vivo*.

## INTRODUCTION

Respiratory syncytial virus (RSV) is an enveloped RNA virus that primarily affects the human respiratory system. It typically causes mild cold-like symptoms in healthy individuals but poses significant risks to infants, the elderly, and those with compromised immune systems (1, 2). RSV infection is widespread, occurring in nearly all young infants and recurring throughout life. Globally, RSV is responsible for 22% of all childhood acute lower respiratory infections, resulting in 66,000 to 199,000 annual deaths in children under the age of five (3, 4). The mortality rate due to RSV infection in developing countries is twice as high as that in developed countries (5). In older adults, RSV infection is associated with severe respiratory tract illness. In the United States, RSV infection results in over 177,000 hospitalizations and 14,000 deaths annually among adults over 65 years old (2).

Efforts to develop an RSV vaccine began in the 1960s but have faced significant challenges, particularly because the formalin-inactivated RSV vaccine induced excessive inflammatory responses, exacerbating bronchiolitis during natural infections (6). Until recently, the U.S. Food and Drug Administration (FDA) has licensed two vaccines based on RSV prefusion F protein (Arexvy and Abrysvo) and an mRNA-based vaccine (mResvia) that encodes the stabilized prefusion F protein. These vaccines have received approval for the prevention of RSV infection in individuals aged 50 years and older or neonates through the active immunization of pregnant individuals. However, they have not yet been approved for other vulnerable populations, including infants and children (7, 8). Recently, the FDA suspended all clinical trials of RSV vaccines in infants and young children following the occurrence of severe illness in infants vaccinated with Moderna’s mRNA-based RSV vaccine.

RSV F is a type 1 transmembrane protein synthesized from the precursor F0 protein, which is composed of 574 amino acids. In the Golgi apparatus, F0 is hydrolyzed by the furin enzyme at two sites (KKRKRR, FCS1; RARR, FCS2), resulting in the release of three fragments: a 27-amino acid polypeptide (pep27), F1, and F2 (9). The F1 and F2 subunits are generated as a heterodimer linked by disulfide bonds (10, 11). Three F1-F2 heterodimers assemble to form a mature RSV F trimer on the cell membrane, at which the trimeric F proteins in a compact prefusion conformation are incorporated onto the envelope of progeny virions (11). The membrane fusion event is initiated once the prefusion F trimer is triggered on the cell surface by an unknown mechanism. Similar to the class I fusion glycoprotein of other enveloped viruses, the prefusion F of RSV undergoes conformational changes during viral fusion with the cell membrane, while the metastable prefusion F is rearranged into an extremely stable postfusion F. This conformational rearrangement can occur spontaneously on the viral envelope or cell membrane and can also be triggered by some stimulation factors, such as cell receptors, high temperature, and low-ionic strength buffer (12–14). RSV F trimer possesses surface antigenic epitopes that can elicit neutralizing antibodies *in vivo* and is the primary target for vaccine-induced protective immunity. The prefusion F of RSV elicits more potent neutralizing antibodies than the postfusion F in human serum. However, constructing a prefusion-stabilized trimeric F is a major challenge for RSV vaccine development due to the conformational instability of the prefusion F trimer, which is readily and irreversibly rearranged into a highly stable postfusion structure on the cell membrane or during the process of protein purification.

Recent progress in RSV vaccine (e.g., Arexvy, Abrysvo, and mResvia) development has been made upon the successful structural biology studies on prefusion-stabilized RSV F. DS-Cav1, the first-generation prefusion F subunit vaccine, provided a structural basis for developing high-potency RSV vaccines. It was designed with two cysteine mutations (disulfide bond [DS]) to form a disulfide and two "cavity-filling" mutations in RSV F (15). These mutations improved the conformational stability of RSV F, whereas the protein yield in cell cultures was limited. Moreover, the purified DS-Cav1 proved unstable during long-term storage (16). A recent study demonstrated that the expression level of DS-Cav1 is significantly lower than Cav1 in HEK293T cells (17), suggesting a negative impact of DS mutation on the protein expression. Removing the DS mutations and introducing two engineered dityrosine crosslinks in DS-Cav1 generated a stable prefusion RSV F vaccine candidate (DT-preF) with high immunogenicity in mice. However, these modifications did not enhance the expression level of DT-preF compared to Cav1 (17).

Other subunit RSV vaccine candidates SC-DM and SC-TM are designed with DM (N67I, S215P) or TM (N67I, S215P, E487Q) mutations and a GS linker between F1 and F2 heterodimers (16). In SC-DM and SC-TM, the furin cleavage site is substituted with GSGSG residues to retain a single-chain (SC) F monomer, preventing the proteolytic exposure of the fusion peptide (FP) and blocking the conformational rearrangements of the prefusion F (16). Previous studies have shown that furin cleavage of pep27 in F0 is crucial for trimerization of F1-F2 heterodimers (18, 19). Consequently, the purified SC-DM and SC-TM partially adopted a trimeric structure in native PAGE and completely shifted to a single-chain (SC) monomer after SDS-PAGE in a non-reduced buffer (16). Therefore, further modifications in SC-DM or SC-TM are suggested to enhance the conformational stability of trimeric prefusion F, which is required for eliciting high-potency neutralizing antibodies *in vivo*.

To construct a prefusion-stabilized F trimer that retains the original antigenic epitopes and immunogenicity, the pre-F-IFLP was designed with four hydrophobic residue substitutions, with both "cavity-filling" and DM mutations. This prefusion-stabilized F construct preserves the original furin cleavage site and disulfide bonds and possesses the characteristics of high thermal stability, high acid-base resistance, and an extended storage life. Notably, compared to DS-Cav1, pre-F-IFLP exhibited higher expression levels in cultured cells and elicited higher serum-neutralization titers in mice, providing full protection against RSV challenge in murine models.

## RESULTS

### Design and identification of mutations to stabilize the prefusion RSV F on the cell membrane

**Fig 1:**
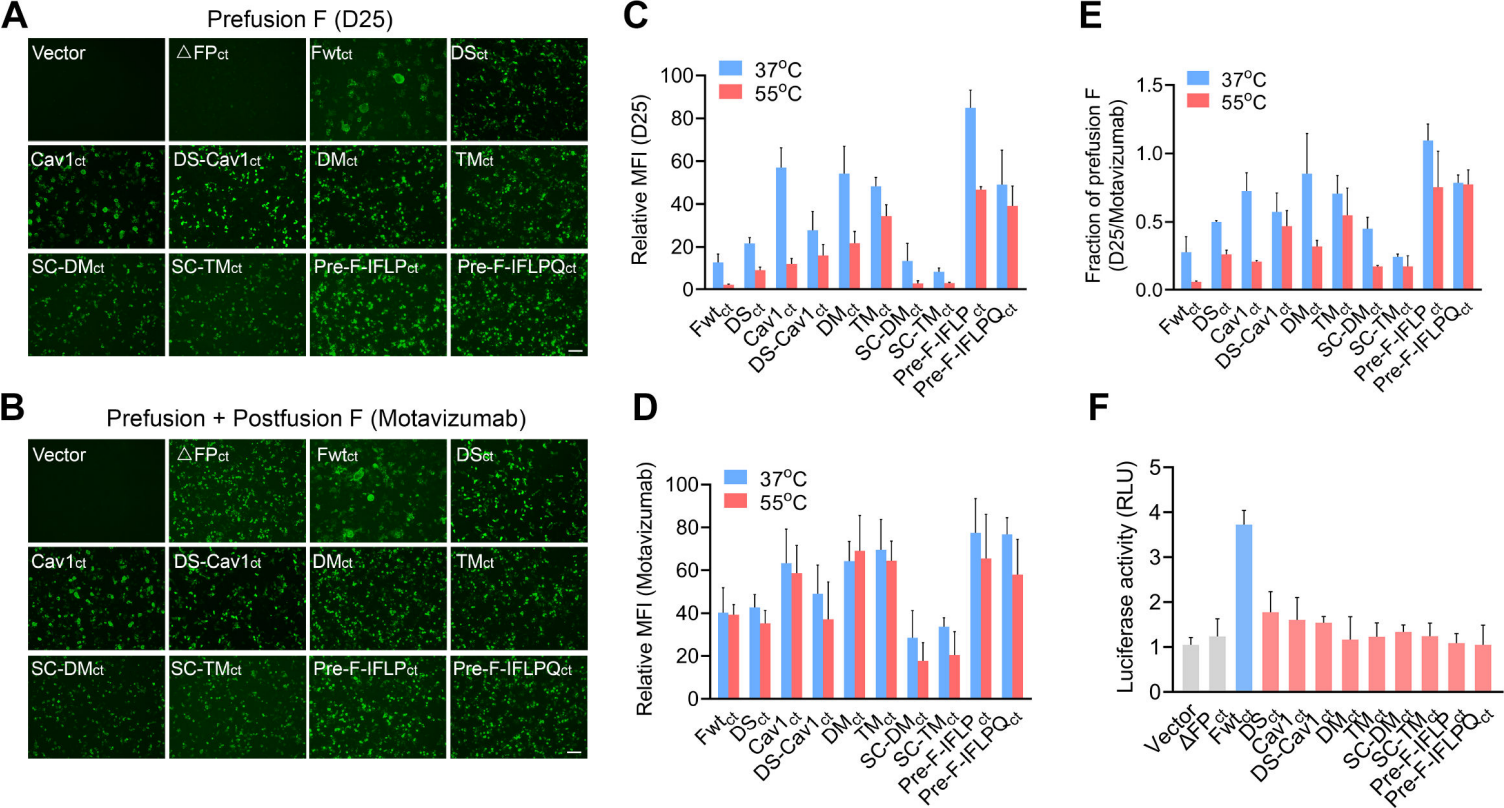
Mutations to stabilize the prefusion RSV F protein. HEK293T cells were transfected with pcDNA3.1 vectors encoding various RSV F variants. Cells were stained with D25 for prefusion F (**A**) and motavizumab for prefusion/postfusion F (**B**), followed by incubation with an Alexa 488-conjugated secondary antibody. Scale bars, 100 µm. (**C, D, E**) Following incubation for 10 min at 37°C or 55°C, the prefusion and postfusion F in cells were determined using flow cytometry. MFI, mean fluorescence intensity. (**F**) Cell-cell fusion activity in transfected cells was assessed using a dual-luciferase reporter assay. (C–F) Data are presented as mean ± SD, *n* = 3 biological replicates.

To construct prefusion-stabilized RSV F with high expression levels in cultured cells, full-length RSV F variants with various amino acid mutations were cloned into the pcDNA3.1 vector and expressed in HEK293T cells (Table S1). As expected, RSV F variants with the 10-amino acid (FLGFLLGVGS) deletion in FP maintained a postfusion conformation (Fig. 1A and B). No detectable prefusion F was observed in F_ΔFP_-transfected cells when stained with D25, a monoclonal antibody specific for prefusion RSV F protein. Compared to the wild-type (WT) F, the DS mutations (S155C, S290C) increased the conformational stability of prefusion F but resulted in decreased protein expression levels (Fig. 1A through E). At 37°C, RSV F constructs with cavity-filling (Cav1) mutations (S190F, V207L) exhibited a 2.6, 1.5, and 1.3-fold higher ratio of prefusion F (D25/motavizumab) on the cell surface than WT F, DS and DS-Cav1 variants, respectively (Fig. 1C through E). Moreover, the total F protein expression level of the Cav1 variant, as stained by motavizumab, is higher than that of WT F, DS, and DS-Cav1 variants. These results suggest that Cav1 mutations are important for stabilizing the prefusion conformation of DS-Cav1 and enhancing its protein expression level in cells.

Subsequently, we observed that SC mutation, which introduced a GS linker between F1 and F2 subunits, did not enhance the protein expression level or improve its stability on the cell membrane (Fig. 1C through E). Combinations of cavity-filling mutation with DM (N67I, S215P) or TM (N67I, S215P, E487Q) resulted in two prefusion-stabilized full-length RSV F variants, pre-F-IFLP_ct_ and pre-F-IFLPQ_ct_. At both 37°C and 55°C, the pre-F-IFLP_ct_ variant, which includes four hydrophobic residue mutations (N67I, S190F, V207L, S215P), substantially increased RSV F expression levels and maintained a more stable prefusion F conformation compared to the other F variants tested in this study (Fig. 1C through E). Compared with wild-type RSV F, all the mutated F variants produced fewer and smaller syncytia in cells. Notably, the pre-F-IFLP_ct_ and pre-F-IFLPQ_ct_ variants exhibited lower membrane fusion activity than the other tested RSV F variants (Fig. 1F).

### Stability assessment of ectodomain RSV F variants

**Fig 2:**
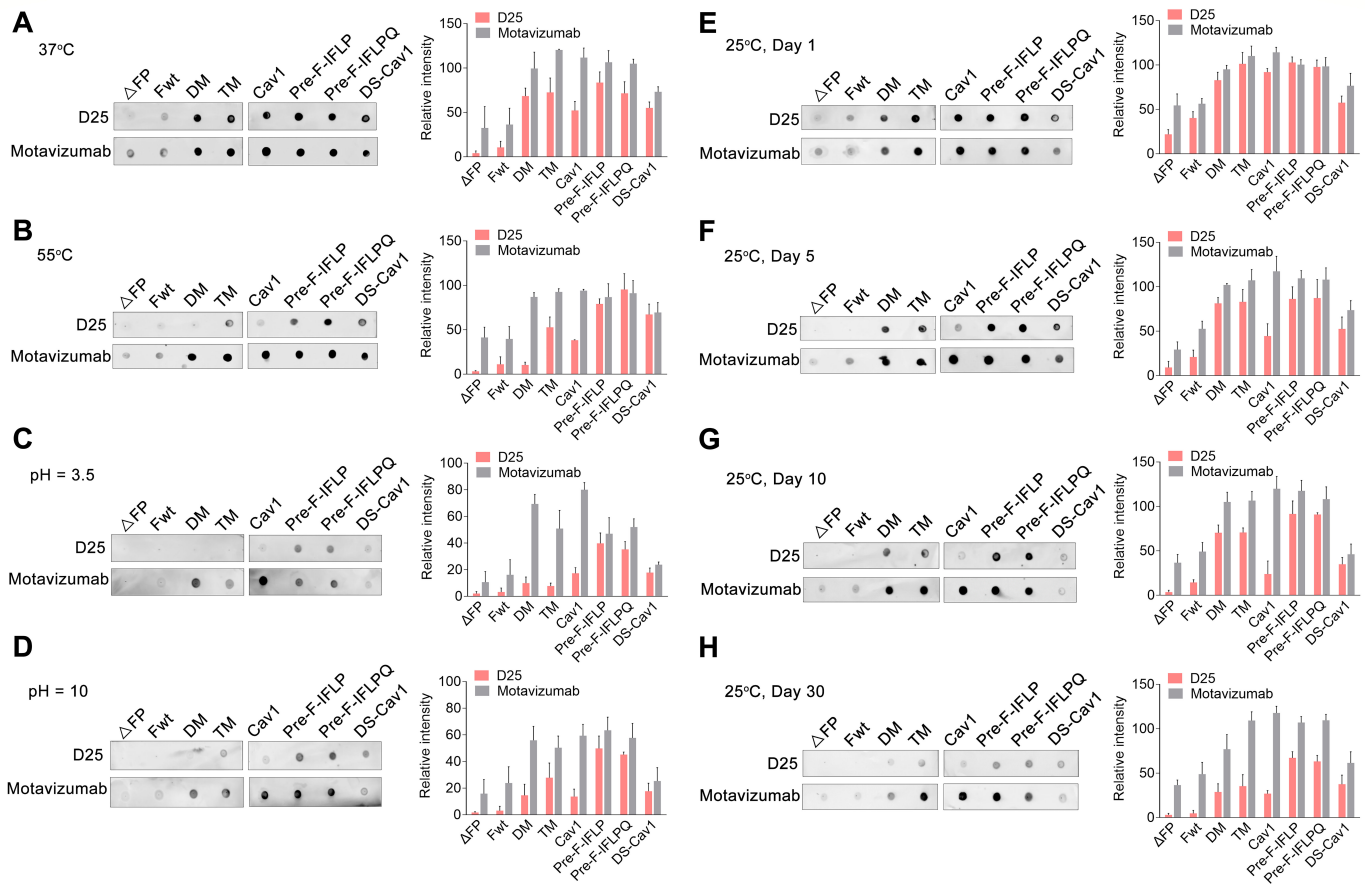
Characterization of the physical stability of the prefusion F variants. (**A, B**) Purified RSV F ectodomains were transferred to a nitrocellulose membrane and incubated for 10 min at 37°C or 55°C. (**C, D**) The RSV F constructs on the nitrocellulose membrane were treated with PBS-HCl (pH 3.5) or PBS-NaOH (pH 10) solution for 30 min at 25°C. (E–H) The nitrocellulose membrane with RSV proteins was stored at 25°C for 1, 5, 10, and 30 days. All RSV F constructs on the nitrocellulose membrane were incubated with D25 and motavizumab, followed by detection with horseradish peroxidase-conjugated secondary antibodies with ECL solution. (A–H) Data are presented as mean ± SD, *n* = 3 biological replicates.

To generate soluble prefusion-stabilized RSV F proteins, the ectodomains of RSV F were engineered with various mutations and fused with T4 fibritin trimerization motif and His tag at C-terminus, then expressed in HEK293T cells. All soluble RSV F constructs were subsequently prepared for physical stability evaluation. Hardly any prefusion F was detected in the ΔFP solution (Fig. 2A and B), consistent with previous reports that soluble RSV F proteins lacking the FP adopted a postfusion conformation (20). At 37°C or 55°C, the prefusion F fraction of pre-F-IFLP was higher than that of Fwt, DM, TM, and DS-Cav1 (Fig. 2A and B). After incubation at pH 3.5 or 10, the prefusion F of DM, TM, Cav1, and DS-Cav1 was largely rearranged to a postfusion conformation (Fig. 2C and D). In contrast, both pre-F-IFLP and pre-F-IFLPQ remained stable at these pH levels. Additionally, most pre-F-IFLP retained in its prefusion conformation after 10 days of storage at 25°C (Fig. 2E through G). Although some prefusion F structures in the pre-F-IFLP solution transitioned to the postfusion conformation after 30 days of storage at 25°C, pre-F-IFLP maintained a higher prefusion fraction compared to other tested F variants (Fig. 2H). In contrast, nearly all F_wt_ changed to postfusion structure after 10 days of storage at 25°C (Fig. 2C and D). Overall, these results suggest that the four hydrophobic residue mutations in pre-F-IFLP substantially enhance its stability in the prefusion conformation.

### Antigenic characterization of the Pre-F-IFLP construct

**Fig 3:**
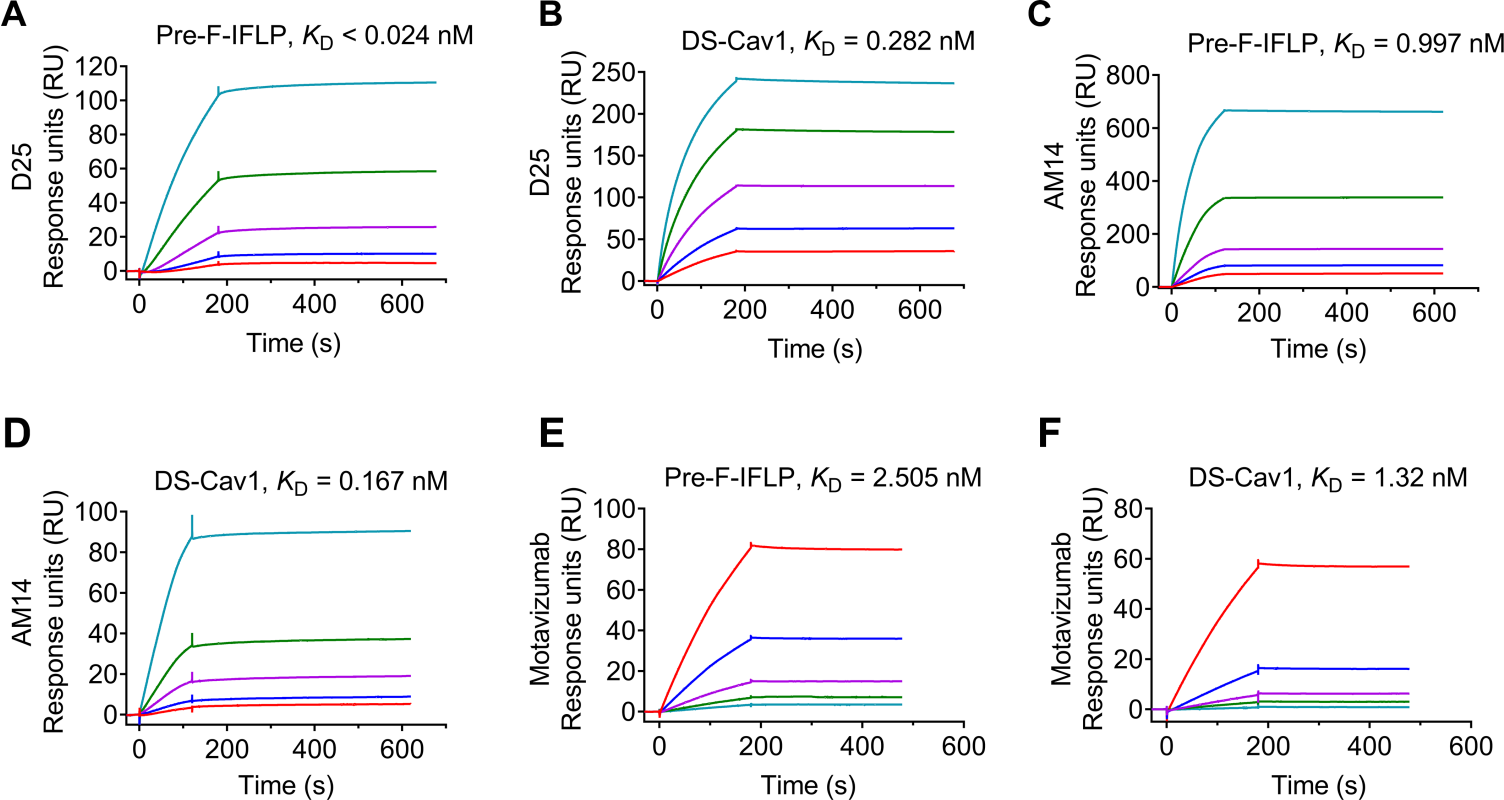
Antigenic characterization of prefusion F variants. Binding curves for D25, AM14, and motavizumab to pre-F-IFLP and DS-Cav1 were generated using surface plasmon resonance assays. RSV F-specific antibodies were covalently immobilized onto the surface of CM5 sensor chips. Soluble recombinant proteins were diluted in PBS and flowed over the chips. Data were analyzed using Biacore S200 Evaluation Software, and the KD values were calculated to assess the binding affinity of RSV F constructs with anti-RSV F-specific antibodies. (**A, C**) Pre-F-IFLP was tested at concentrations of 3.125, 6.25, 12.5, 25, and 50 nM; (**B**) DS-Cav1 at 12.5, 25, 50, 100, and 200 nM; and (**D, E, F**) both pre-F-IFLP and DS-Cav1 were tested at 6.25, 12.5, 25, 50, and 100 nM.

Purified pre-F-IFLP and DS-Cav1 maintain a trimeric conformation, as demonstrated by native-PAGE and native-western blot (WB) analysis (Fig. S1A). Next, the trimeric RSV F was collected via size exclusion chromatography (Fig. S1B). Although some pre-IFLP and DS-Cav1 trimers were dissociated into monomers after SDS-PAGE and SDS-WB under non-reduced conditions, the trimeric forms of pre-F-IFLP and DS-Cav1 still displayed a higher band intensity compared to their monomers (Fig. S1C). No monomers and trimers were detected in pre-F-IFLP and DS-Cav1 after denaturing SDS-WB with dithiothreitol, suggesting that both disulfide bonds in pre-F-IFLP and DS-Cav1 were completely reduced (Fig. S1C). Surface plasmon resonance (SPR) assays were subsequently conducted to detect the binding affinity of pre-F-IFLP and DS-Cav1 to neutralizing antibodies. Pre-F-IFLP showed a strong binding affinity to the D25 antibody. The dissociation equilibrium constant (KD) for D25 binding to pre-F-IFLP and DS-Cav1 was <0.024 and 0.282 nM, respectively (Fig. 3A and B), suggesting a stronger affinity of D25 with pre-F-IFLP than with DS-Cav1. In addition, the KD values for pre-IFLP binding to AM14 or motavizumab were at the nanomolar levels comparable to those observed for DS-Cav1 with the two neutralizing antibodies (Fig. 3C through F). Taken together, these results suggest that the purified pre-F-IFLP maintains a trimeric prefusion conformation with highly potent antigenic epitopes recognized by prefusion F-specific neutralizing antibodies.

### Neutralizing efficacy of sera from pre-F-IFLP-immunized mice against RSV F-mediated infection

**Fig 4:**
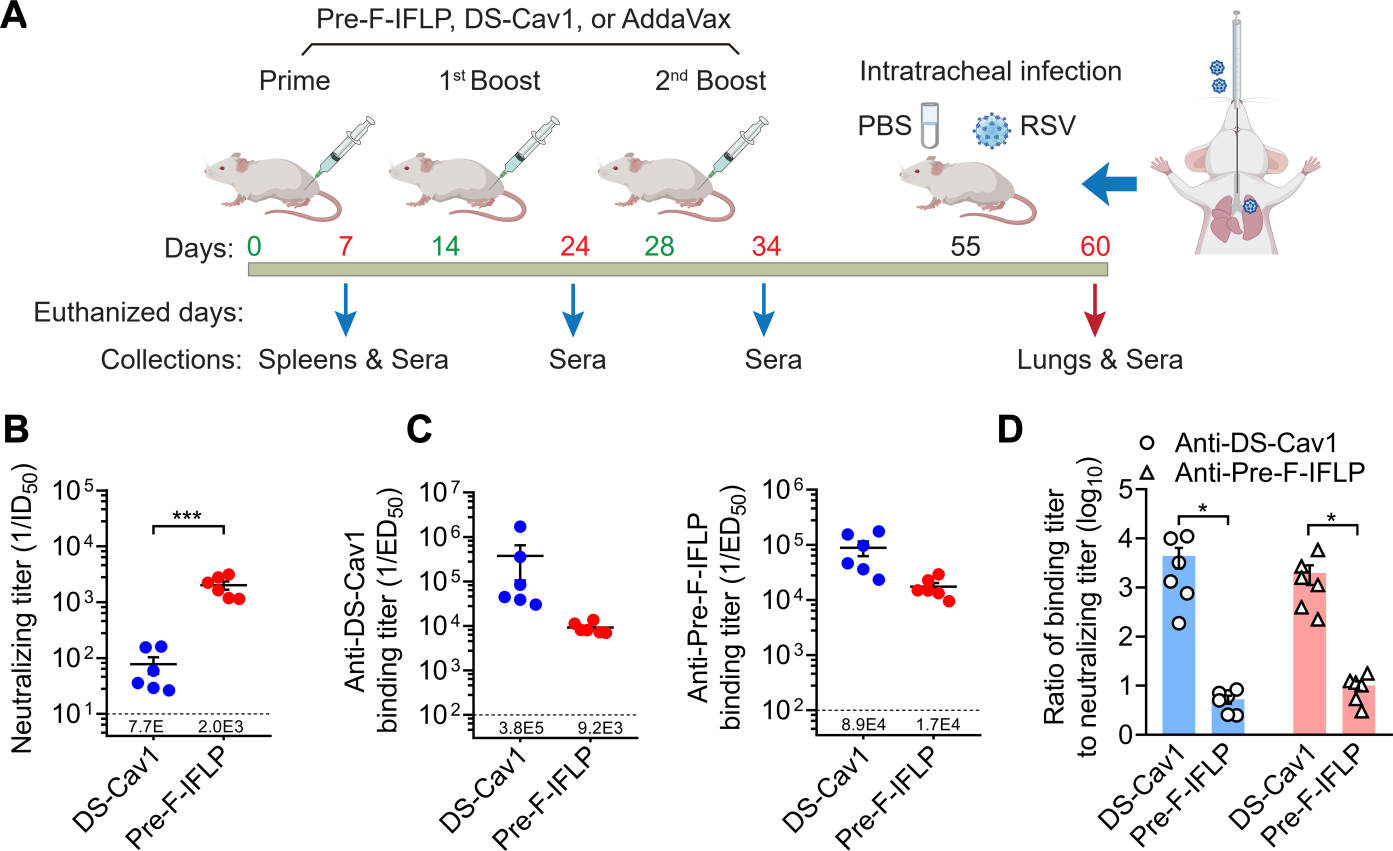
Neutralizing efficacy of mouse serum after first boost immunization. (**A**) Schematic showing the schedule of animal studies. (**B**) The mouse serum after twice immunization was collected on day 24 and prepared for detection of neutralizing titer. Dilutions of the mouse serum to neutralize 50% of the RSV infection (ID_50_) were measured. (**C**) Following the first boost immunization, antibody titers in the mouse serum were detected. (**D**) Ratio of binding titers to neutralizing titers derived from the data in (**B**) and (**C**). Each dot represents the ratio of ED_50_/ID_50_ for each mouse. (B–D) Each dot represents individual mice. Data are mean ± SEM, *n* = 6. An unpaired two-tailed *t*-test was used to measure the statistical differences between the compared groups. *, *P* < 0.05; ***, *P* < 0.001.

To evaluate the *in vivo* immunogenicity of pre-F-IFLP and its protective efficacy to prevent RSV infection, BALB/c mice were vaccinated with the pre-F-IFLP or DS-Cav1 formulated with AddaVax adjuvant. On days 14 and 28, the mice received first and second boost immunizations, respectively (Fig. 4A). Seven days after the prime immunization, immunoregulatory factors in the mouse serum were detected using enzyme-linked immunosorbent assay (ELISA). Mice immunized with pre-F-IFLP exhibited a slight increase in the secretion level of interferon γ (IFN-γ) (Fig. S2A), while other proinflammatory or immunoregulatory factors remained comparable to those in the AddaVax control group (Fig. S2B through D). Furthermore, flow cytometry analysis revealed that pre-F-IFLP immunization led to an increased proportion of CD4^+^CD44^+^IL4^+^cells in spleen lymphocytes without altering the proportion of CD8^+^CD44^+^IL4^+^cells (Fig. S2F and G). These results suggest that pre-F-IFLP presumably activates the Th1-associated immune responses.

Following two immunizations with either pre-F-IFLP or DS-Cav1, neutralizing antibodies were generated in the mouse serum and showed strong binding affinity to their respective immunogens. Sera from both pre-F-IFLP- and DS-Cav1-immunized mice significantly neutralized RSV infection. Notably, the neutralizing antibodies induced by pre-F-IFLP after the first boost immunization had a 26-fold higher potency in preventing RSV infection compared to those induced by DS-Cav1 (Fig. 4B). Compared with DS-Cav1 immunization, the neutralizing antibodies in the serum of pre-F-IFLP-immunized mice exhibited lower binding titers to soluble prefusion RSV F constructs (Fig. 4C). Furthermore, the ratio of the binding titer to the neutralizing titer in pre-F-IFLP-immunized mice was significantly lower than that observed in DS-Cav1-immunized mice (Fig. 4D). These results suggest that the neutralizing antibodies induced by pre-F-IFLP immunization are more effective in reducing RSV infection than those generated by DS-Cav1 immunization.

**Fig 5:**
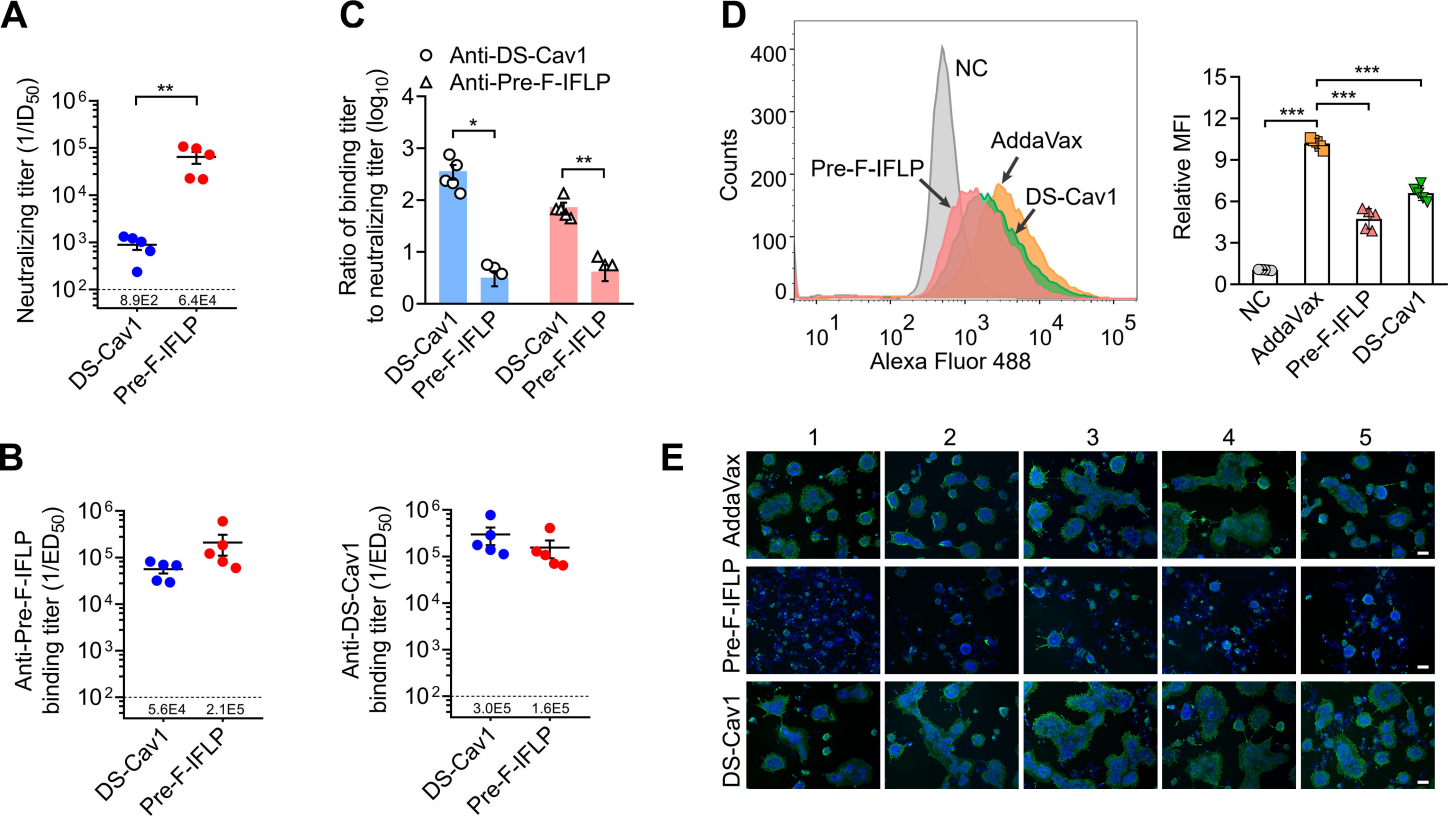
Neutralizing efficacy of mouse serum after second boost immunization. (**A**) On day 34, mouse serum was collected following three rounds of immunization and prepared for analysis of neutralizing efficacy. Neutralizing titers of the mouse serum to inhibit RSV infection were determined. ID_50_ is the serum dilutions required to reduce RSV infection by 50%. (**B**) Binding titers of neutralizing antibodies in the mouse serum to respective immunogens. (**C**) Ratios of binding titers to neutralizing titers were calculated. (**D**) The ectodomain of RSV F (amino acids 1–526) was expressed, stored in a high-ionic strength buffer (20 mM Tris, 500 mM NaCl, pH 7.4, 10% glycerinum), and then pre-incubated with sera from immunized mice. The mixtures were diluted in PBS and subsequently incubated with HEp-2 cells for 1 h. Surface binding of the RSV F ectodomains to HEp-2 cells was stained using motavizumab and Alexa Fluor 488-conjugated secondary antibody and subsequently detected by flow cytometry. Mean fluorescence intensity (MFI) was analyzed using FlowJo v.10. (**E**) HEK293T cells were transfected with plasmids encoding full-length RSV F and then treated with diluted sera from immunized mice. Syncytia and cell nuclei were stained with RSV F-specific antibody and 4′,6-diamidino-2-phenylindole, respectively. Bar, 100 µm. (A–D) Data are presented as mean ± SEM, *n* = 5. An unpaired two-tailed *t*-test was used for comparisons between two groups. *, *P* < 0.05; **, *P* < 0.01; ***, *P* < 0.001.

In comparison to the first boost immunization, the second boost immunization substantially enhanced the neutralizing capacity of mouse serum against RSV infection. Following two boost doses of pre-F-IFLP, the average serum neutralizing titers in vaccinated mice reached levels of 64,000, nearly 32-fold higher than those in mice that received only the first boost immunization (Fig. 5A and B). Furthermore, the serum binding titers to prefusion F immunogens in pre-F-IFLP-immunized mice markedly increased following the second boost immunization (Fig. 5B and C), indicating that the second boost immunization elicited a greater abundance of neutralizing antibodies. The serum neutralizing titers in pre-IFLP-immunized mice were 72-fold higher than those in mice immunized with DS-Cav1 (Fig. 5A). Importantly, neutralizing antibodies induced by pre-F-IFLP demonstrated a stronger efficacy in inhibiting RSV infection, as evidenced by significantly lower ratios of binding titer to neutralizing titers in sera from pre-F-IFLP-immunized mice compared to those from DS-Cav1-immunized mice (Fig. 5C).

During the initial stage of RSV infection, the viral F protein interacts with the cell membrane and then mediates the virus-cell membrane fusion. The soluble ectodomains of RSV F protein in a high-molarity buffer adopt a pretriggered conformation and are capable of interacting with the cell membrane (10, 21, 22). Upon the pretreatment with diluted serum from AddaVax-immunized mice, a large fraction of soluble F(ecto) adsorbed onto the cell membrane. However, in the presence of the diluted sera from pre-F-IFLP- or DS-Cav1-immunized mice, the amount of F(ecto) on the cell membrane was significantly decreased. Pre-F-IFLP vaccination induced more potent neutralizing antibodies than DS-Cav1 to block the interaction of F(ecto) with the cell membrane (Fig. 5D).

We next determined whether the cell-cell fusion and syncytia formation could be inhibited by the serum from vaccinated mice. In this assay, HEK293T cells were transfected with plasmids encoding full-length RSV F protein and treated with the diluted sera from pre-F-IFLP- or DS-Cav1-immunized mice. Treatment with serum from pre-F-IFLP-immunized mice resulted in a nearly complete inhibition of the formation of large multinucleated syncytia (Fig. 5E). In contrast, cells treated with serum from DS-Cav1-immunized mice displayed numerous multinucleated syncytia, with only a slight decrease in syncytia numbers. Collectively, these results suggest that pre-F-IFLP immunization elicited high titers of RSV-neutralizing antibodies, the neutralizing efficacy of which is more potent than the antibodies induced by DS-Cav1.

### Preventive effects of pre-F-IFLP immunization against RSV infection in mice

**Fig 6:**
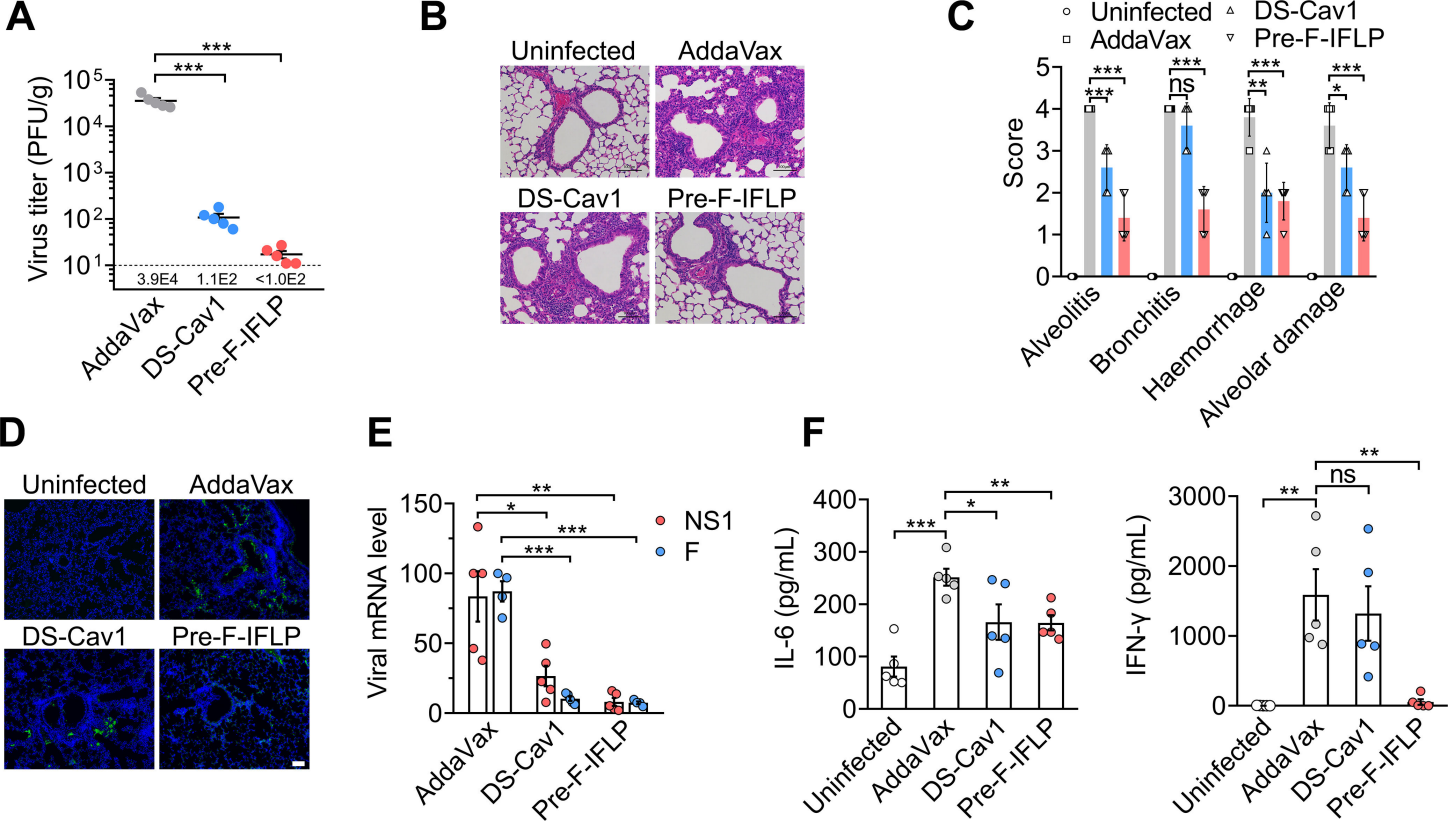
Protective effects of pre-F-IFLP and DS-Cav1 immunizations against RSV infection in mice. (**A**) Mice were intratracheally infected with RSV following three immunizations with either pre-F-IFLP or DS-Cav1. On day 5 postinfection (d.p.i.), viral loads in the lung homogenates of immunized mice were determined. (**B**) Representative hematoxylin and eosin staining images of lung tissue sections were assessed. Scale bars, 100 µm. (**C**) Pathological changes in lung tissues were scored, and representative features of lung lesions were measured. (**D**) Representative immunostaining images of RSV F antigens in lung tissues are shown. Scale bars, 100 µm. (**E**) The mRNA levels of viral NS1 and F genes were detected by RT-PCR. (**F**) On day 5 d.p.i., secretion levels of IL-6 and IFN- γ in mouse serum were measured by ELISA. (**A, C, E, F**) Each dot represents an individual mouse. Data are presented as mean ± SEM, *n* = 5. An unpaired two-tailed *t*-test was used for comparisons between two groups. *, *P* < 0.05; **, *P* < 0.01; ***, *P* < 0.001. ns, no significant difference.

To assess the protective effects of prefusion F vaccination against RSV infection, mice were intratracheally inoculated with RSV A2 following the second boost immunization. Lung tissues of mice were collected 5 days postinfection. Compared to AddaVax-immunized mice, viral titers were reduced by 328-fold in DS-Cav1-immunized mice and by 2,068-fold in pre-F-IFLP-immunized mice (Fig. 6A). Severe inflammatory infiltrations, including bronchitis, alveolitis, alveolar damage, and crushed debris accumulation around bronchioles, were observed in RSV-infected mice (Fig. 6B). In pre-F-IFLP-immunized mice, these pathological changes and inflammatory responses, such as bronchitis, hemorrhage, and alveolitis, were apparently alleviated compared with those in AddaVax-immunized mice (Fig. 6C). Further evidence from immunostaining and RT-PCR assays revealed a marked reduction of viral antigens within the lung tissues of pre-F-IFLP-immunized mice (Fig. 6D), in which the transcript levels of viral NS1 and F genes were suppressed to nearly undetectable levels (Fig. 6E). Both DS-Cav1- and pre-F-IFLP-vaccinated mice exhibited a significant decrease in the serum levels of pro-inflammatory cytokine interleukin 6 (IL-6) (Fig. 6F). Moreover, the secretion level of IFN-γ, an antiviral and inflammation mediator, was significantly suppressed by pre-F-IFLP-immunization. In contrast, DS-Cav1 immunization only slightly reduced IFN-γ levels in mouse serum. Consistent with these findings, RT-PCR analysis further confirmed that pre-F-IFLP immunization significantly reduced the transcription levels of proinflammatory cytokines (IFN-γ, TNF-α, IL-6) in the lung tissues compared to AddaVax immunization (Fig. S3).

## DISCUSSION

The past few years have witnessed significant breakthroughs in RSV vaccine development. GSK's leading vaccine (Arexvy), Pfizer's maternal vaccine (Abrysvo), and Moderna’s mRNA-based vaccine (mResvia) have recently been licensed to prevent RSV infection in older adults or neonates via maternal immunization. However, enrollment for all clinical trials of RSV vaccines targeting infants is now on hold due to safety concerns. In addition, immunization with Arexvy in frail or older people over 80 years old has shown only 14 and 34% efficacy, respectively (17), with no statistical significance observed in clinical studies (21). Maternal vaccination with Abrysvo demonstrated a protective effect comparable to passive immunoprophylaxis with monoclonal antibodies in infants (7). Recently, Moderna reported that mResvia showed only 50% efficacy in preventing RSV infection after 18 months. These clinical outcomes highlight the ongoing medical demand, particularly for high-risk groups, such as infants and immunocompromised populations.

DS-Cav1 is a soluble trimeric RSV prefusion F protein commonly used as a standard reference for RSV vaccine studies. It is designed to stably expose the antigenic site Ø on the apex of prefusion F, which is highly sensitive to prefusion-specific neutralization. Nevertheless, DS-Cav1 is unstable during long-term storage and has a low protein expression level in cultured cells (16, 23). Recent studies have shown that the introduction of a disulfide bond between F1 and F2 subunits increased the stability of prefusion F conformation but reduced the protein yields in cells (17, 23). The vaccine candidates SC-DM and SC-TM were developed by deleting a furin cleavage site and introducing DM or TM mutation with a GS linker between F1 and F1. Some prefusion F trimers were observed in purified SC-DM and SC-TM solutions after native-PAGE, but they were reverted to monomers by SDS-PAGE in a non-reduced buffer (16). This suggests that trimeric SC-DM and SC-TM were stabilized by non-covalent interactions of protein chains, with no disulfide bonds formed between the monomers.

In this study, we investigated the expression levels and conformational stability of RSV F variants containing different mutations derived from DS-Cav1 and SC-DM or SC-TM. Full-length RSV F variants with transmembrane (TM) domain and cytoplasm (CP) tail were utilized to mimic the native trimeric conformation of the RSV F protein. Our results demonstrated that the F variant with DS mutation exhibited greater stability on the cell membrane compared to the wild-type (WT) F or Cav1 variant, as evidenced by a higher fraction of prefusion F in DS variant-transfected cells at 55℃. The Cav1 variant exhibited a higher expression level than DS-Cav1 but displayed lower thermal stability. Consistent with previous reports, the DM and TM mutations greatly increased the protein expression level. Importantly, the combined mutation of Cav1 with DM generated a prefusion-stabilized F variant, pre-F-IFLP, which greatly enhanced the protein yields and conformational stability of prefusion F. With the removal of TM and CP and the introduction of the T4 fibritin trimerization motif (foldon) at the C-terminus, the soluble pre-F-IFLP and pre-F-IFLPQ were stable at 25°C for 30 days, suggesting a wide temperature range for storage. Furthermore, the binding affinity of pre-F-IFLP to the site Ø-specific antibody was over 11.7-fold greater than that of DS-Cav1. Following the first and second boost immunizations in mice, pre-F-IFLP elicited antibody binding titers in the mouse serum that were lower or comparable to those elicited by DS-Cav1. However, the neutralizing antibodies generated by pre-F-IFLP immunization exhibited significantly greater efficacy in reducing RSV infection compared to those induced by DS-Cav1. In addition to the Ø apex sites, there may exist a more potent neutralizing epitope on the trimeric prefusion F that is specifically targeted by pre-F-IFLP-induced antibodies.

Minimal lesions and inflammatory cell infiltrations were observed in the lung tissues of pre-F-IFLP-vaccinated mice. Notably, the four mutations in pre-IFLP are all hydrophobic amino acid substitutions, suggesting a critical role of the hydrophobic effect within the central cavity of the prefusion F trimer. This is important for maintaining its conformational stability and facilitating the exposure of key antigenic sites, leading to a strong and sustained immune response, ultimately contributing to comprehensive protection against RSV infection. It is noted that DS-Cav1 has been reported in several studies to be more effective than observed in the present study in eliciting neutralizing antibody responses and protecting against RSV infection. These discrepancies can be attributed to variations in animal models, adjuvants, dosages, and time points for measurement. In our study, compared to DS-Cav1, pre-IFLP exhibited significantly higher prefusion conformational stability, elevated neutralizing titers, and greater potency in preventing RSV infection in mice, highlighting its potential as a promising candidate for incorporation into various vaccine platforms, including mRNA-based and viral vector vaccines.

Previous reports have shown that most RSV F vaccines are designed by amino acid mutations with the introduction of covalent linkages (e.g., disulfide bonds, dityrosine crosslinks, or GS linkers) to stabilize prefusion F conformation or enhance protein expression. Some of these mutations effectively prevent the rearrangement of prefusion F while maintaining relatively high protein expression levels. Nevertheless, the introduction of such external bonds may impede the trimerization of F1-F2 heterodimers, potentially resulting in the loss of original antigenic epitopes. Recent efforts have explored alternative strategies for stabilizing prefusion RSV F and assessing their *in vivo* immunogenicity. Second-generation DS2-F glycoprotein immunogens developed through iterative structure-based design elicited higher neutralizing titers than DS-Cav1 in mice (24). Prefusion RSV F stabilization can be achieved by inhibiting localized structural transitions that precede large-scale conformational rearrangements (25). Additionally, the foldon-free prefusion F trimer vaccine minimizes off-target immune responses (26). Recently, an uncleaved prefusion vaccine in a native-like, closed trimeric conformation was designed without the incorporation of any interprotomer disulfide bonds (27). Our study presents an alternative example for stabilizing the trimeric prefusion F and enhancing protein expression through hydrophobic amino acid substitutions. Further structural biology investigations are required to elucidate the molecular mechanism by which these hydrophobic amino acid substitutions influence the conformational stability of RSV F and its immunogenicity *in vivo*.

## MATERIALS AND METHODS

### Cells and virus

The human epithelial type 2 cell line (HEp-2) and human embryonic kidney cell line (HEK293T) were purchased from the American Type Culture Collection (ATCC). HEp-2 cells were cultured in Dulbecco’s modified Eagle’s medium supplemented with 10% fetal bovine serum (FBS). The RSV A2 strain (VR-1540; ATCC) was propagated in HEp-2 cells, and viral titers were detected using the TCID_50_ assay.

### Immunofluorescence assay

HEK293T cells were seeded into 24-well plates 1 day before transfection. After incubation for 24 h, the cells were transfected with plasmids encoding various RSV F variants using Lipofectamine 2000 transfection reagent. At 5 h post-transfection, the Opti-MEM medium was replaced with fresh cell growth medium. At 48 h post-transfection, the cell culture supernatant was discarded, and the cells were washed twice with PBS. The RSV F proteins in the cells were then detected using humanized monoclonal antibody D25 (PABL-322; Creative Biolabs) or motavizumab (TAB-709; Creative Biolabs) for 2 h at room temperature (RT), followed by staining with Alexa Fluor 488-conjugated anti-human secondary antibody (A11013; Thermo Fisher Scientific) for 1 h at RT. After washing with PBS, the cells were visualized and photographed under a fluorescent microscope.

### Cell-surface triggering assay

The pcDNA3.1 plasmids encoding full-length wild-type RSV F or RSV F variants were transfected into HEK293T cells using the Lipofectamine 6000 Kit (C0526, Beyotime). At 48 h post-transfection, the cells were collected and heat-shocked at 55℃ for 10 min and then stained with D25 or motavizumab for 2 h at RT, followed by incubation with Alexa Fluor 488-conjugated goat anti-human secondary antibody for 1 h at RT. After washing three times with PBS, fluorescence intensity was measured using a BD Biosciences flow cytometer. The data were analyzed using FlowJo v.10.

### Cell-cell fusion assay

A dual-luciferase assay was performed to quantify intercellular membrane fusion mediated by RSV F proteins. HEK293T cells were seeded into 24-well plates and incubated for 24 h. One group of cells was transiently co-transfected with plasmids expressing RSV F variants and pT7-luc, a plasmid containing the firefly luciferase under the control of T7 RNA polymerase. Another group of cells was transfected with pRL-TK, which encodes Renilla luciferase, and pCAG-T7 Pol, a plasmid expressing T7 RNA polymerase. The two groups were mixed in a 1:1 ratio 24 h post-transfection and then seeded into 96-well plates. In fused cells, the T7 RNA polymerase drives the expression of firefly luciferase, correlating with cell-cell fusion activity. At 72 h post-transfection, cellular luciferase activity was quantified using the Dual-Luciferase Reporter Assay System (Promega, WI, USA).

### Expression and purification of RSV F proteins

HEK293T cells were transiently transfected with pcDNA3.1 vectors encoding the ectodomains of RSV F (amino acids 1–513) and its variants with various mutations using Lipofectamine 2000 Transfection Reagent. At 5 days post-transfection, cell supernatants containing soluble RSV F constructs were collected and centrifuged at 2,000 rpm for 10 min to remove cell debris. The supernatants were passed over a Ni-NTA column and eluted with 300 mM imidazole in a buffer containing 50 mM Tris-HCl and 150 mM NaCl (pH 8.0). The eluted fractions were concentrated and further purified by size-exclusion chromatography using a Superdex 200 column (GE Healthcare). The purified proteins were analyzed by SDS-PAGE under reducing or non-reducing conditions, transferred to PVDF membranes, and stained with motavizumab. After incubation at 4°C for 12 h, the membranes were washed three times with PBST (0.05% Tween 20 in PBS) and then incubated with a horseradish peroxidase (HRP)-conjugated secondary antibody for 1 h at RT. The reaction was completed with a chemiluminescent agent, and detection was performed using a chemiluminescence imager (Amersham Imager 600, GE Healthcare).

### Dot blot assay

Soluble RSV F and F variants were blotted onto nitrocellulose membranes, dried under RT, and then blocked with a 5% w/v bovine serum albumin (BSA) solution in PBS for 2 h. To assess the physical stability of the RSV F constructs, the nitrocellulose membranes were subjected to different stress conditions, including low or high pH (3.5 or 10), elevated temperature (55°C), and extended storage periods (1–30 days) at RT. After washing thrice with PBS, the membranes were incubated with D25 or motavizumab, followed by staining with HRP-conjugated secondary antibodies. The membranes were then washed with PBS, incubated with ECL solution, and visualized using a chemiluminescence imager (Amersham Imager 600; GE Healthcare). The integrated density values of each blot were calculated using ImageJ software.

### Surface plasmon resonance assay

To assess the binding affinity of pre-F-IFLP and DS-Cav1 with RSV F-specific antibodies, surface plasmon resonance assays were conducted at 20°C using a Biacore S200 instrument. RSV F antibodies (D25, motavizumab, and AM14) were diluted in 10 mM sodium acetate buffer and immobilized onto the surface of CM5 sensor chips. A regeneration solution was injected to remove non-covalent bound antibodies from the chips. A blank control with no antibody on the sensor chip surface was used as a reference. Soluble purified RSV F proteins were prepared in serial dilutions with PBS and then flowed over the different sensor channels at a flow rate of 30 µL/min. Binding responses between the antibodies and analyte proteins were recorded by the SPR system, and data were processed using Biacore S200 Evaluation Software.

### Immunization

All animal experiments were conducted under the ethical approval of the Institutional Animal Care and Use Committee of Jinan University and in compliance with relevant ethical regulations. Six-week-old female BALB/c mice were purchased from Sipeifu, Biotechnology Co., Ltd. (Beijing, China). The mice were randomly divided into different experimental groups, with 24 mice per group. Before immunization, immunogens were gently mixed at a 1:1 vol/vol ratio with AddaVax adjuvant (vac-adx-10; InvivoGen) to reach a final antigen concentration of 0.05 mg/mL. On days 0, 14, and 28, all mice received intramuscular injections of 50 µL AddaVax or immunogens in AddaVax into the gastrocnemius muscle. Blood samples were collected via cardiac puncture on days 7, 24, and 34, and the sera were prepared for the detection of neutralizing and antibody binding titers and inflammatory or immunoregulatory cytokines. The lymphocyte immunophenotype in the spleens of the mice was determined using flow cytometry. On day 27 after the second boost immunization, five mice in each group were intratracheally infected with 10^5^ plaque-forming units (PFU) of RSV in PBS or an equal volume of PBS. The mice were euthanized on day 5 postinfection. Lung tissues and sera of mice were collected and prepared for evaluation of vaccine efficacy.

### Virus neutralization

The neutralizing effect of mouse sera on RSV infection was assessed using a TCID_50_ assay. Serial dilutions of sera from immunized mice were pre-incubated with virus suspensions for 30 min at 37°C, then added to HEp-2 cells in 96-well plates. Control cells were inoculated with the virus without pre-treatment of sera. At 48 h.p.i., virus suspensions from each well were collected to determine RSV titer using the TCID_50_ assay.

### Enzyme-linked immunosorbent assay

To determine the binding titers of neutralizing antibodies in the mouse serum to DS-Cav1 or pre-F-IFLP, Maxisorp (Nunc) ELISA plates were coated overnight at 4°C with 0.1 µg/well of antigens (DS-Cav1 or pre-F-IFLP). The plates were then blocked with 4% BSA in PBS at RT for 1 h. Serial dilutions of mouse serum were added to each well and incubated for 2 h at RT. After washing with PBS, an anti-mouse IgG antibody conjugated with alkaline phosphatase (Jackson Immunoresearch) for mouse sera was added. The plates were washed three times and reacted with p-nitrophenyl phosphate substrate for 20 min at RT. The absorbance at 405 nm was measured using a microplate reader. To determine the levels of inflammatory cytokines, mouse serum was collected on day 7 after the prime immunization and 5 days post-RSV infection. Sera and standards were serially diluted and added to ELISA plate wells, followed by incubation for 2 h at RT. The plates were washed and then incubated with the biotin-conjugated antibodies specific for IL-6, IFN-γ, TNF-α, IL-2, or IL-4. After incubation for 1 h at RT, streptavidin-HRP solution was added into each well and incubated for 30 min. The cells were then washed three times with PBS and incubated with the TMB substrate solution. The reaction was stopped after 5–30 min by adding an equal amount of stop solution. Absorbance at 450 nm was detected using a microplate reader.

### Flow cytometry

Spleen lymphocytes were collected from mice at 7 days after prime immunization and cultured in RPMI 1640 medium supplemented with 10% (v/v) FBS, 100 U/mL penicillin, and 100 µg/mL streptomycin for 72 h. The cells were activated by adding DS-Cav1 or pre-F-IFLP at a concentration of 1 µg/mL. Subsequently, the activated splenocytes were treated with Brefeldin A (BD Biosciences) to enhance the intracellular cytokine staining signals. After washing with PBS, the cells were stained with anti-CD8, anti-CD4, and anti-CD44 antibodies (all from BioLegend) for 20 min. The cells were then fixed and permeabilized to allow for intracellular staining with anti-IFN-γ and anti-IL-4 antibodies (all from BioLegend). Flow cytometry data were acquired using a NovoCyte flow cytometer (ACEA Biosciences) and analyzed using FlowJo V10 software.

### Lung histopathology

The lungs of mice were harvested at 5 d.p.i., fixed in 4% paraformaldehyde solution, and subsequently embedded in paraffin, sectioned, and stained with hematoxylin and eosin (H&E). The severity of alveolitis, bronchitis, hemorrhage, and alveolar damage was arbitrarily assessed using a four-tiered scoring system: 0 (negative), 0–1 (mild), 1–2 (moderate), 2–3 (moderately severe), and 3–4 (severe).

### Statistical analysis

Data are presented as mean ± standard deviation (SD) or standard error of the mean (SEM). Statistical analyses were performed using GraphPad Prism, version 9.4.1 (GraphPad Software, Inc., La Jolla, CA, USA). Statistical differences between groups were determined using a two-tailed student’s *t*-test, with a *P*-value < 0.05 considered statistically significant.

## Data Availability

All data needed to evaluate the conclusions in the paper are present in the paper and the supplemental material.

## SUPPLEMENTAL MATERIAL

Supplemental material jvi.00087-25-s0001.pdf Figures S1 to S3; Table S1.

ASM does not own the copyrights to Supplemental Material that may be linked to, or accessed through, an article. The authors have granted ASM a non-exclusive, world-wide license to publish the Supplemental Material files. Please contact the corresponding author directly for reuse.
