# Supplementary material for: Hydrophobic residue substitutions enhance the stability and in vivo immunogenicity of respiratory syncytial virus fusion protein
Source: J Virol. 2025 May 28;99(6):e00087-25. doi: 10.1128/jvi.00087-25 (PMC12172430; doi:10.1128/jvi.00087-25)
Supplement: Supplemental material — Figures S1 to S3; Table S1. [file jvi.00087-25-s0001.pdf]

# Supplementary Materials for

## **Hydrophobic residue substitutions enhance the stability and *in vivo* immunogenicity of respiratory syncytial virus fusion protein**

Qiaoyun Song *et al.*

*Corresponding author. Email: tangw@jnu.edu.cn (Wei Tang), wangying_cpu@163.com (Ying Wang), chywc@aliyun.com (Wencai Ye)

**This PDF file includes:**

Figures. Supplementary Fig.1 to Fig.3

Tables. Supplementary Table 1

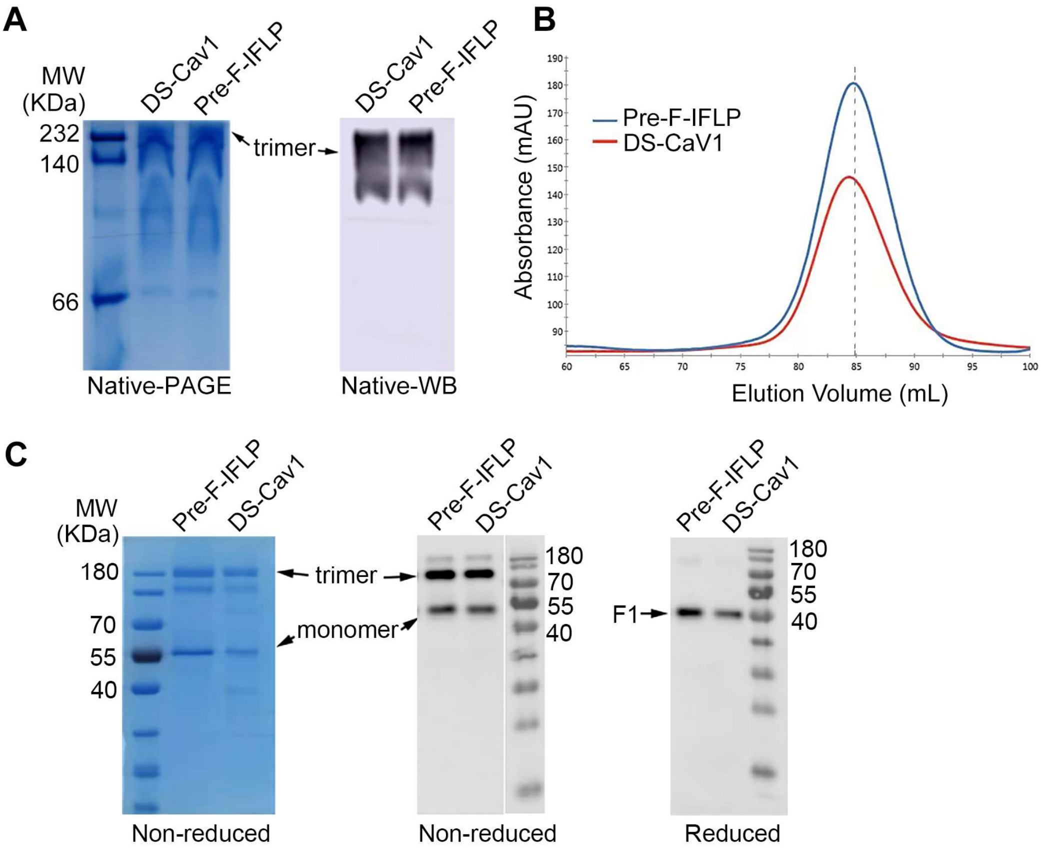


**Supplementary Fig. 1. Conformational states of pre-F-IFLP and DS-Cav1.** (**A**) Purified pre-F-IFLP and DS-Cav1 were analyzed by Native-PAGE, followed by Native-Western blot (WB) using specific anti-RSV F antibody. (**B**) Size-exclusion chromatography (SEC) analysis of soluble pre-F-IFLP and DS-Cav1. (**C**) SDS-PAGE (left panel) without dithiothreitol (DTT) and SDS-WB without (middle panel) or with (right panel) DTT, were conducted to assess the trimeric and monomeric forms of RSV F constructs.

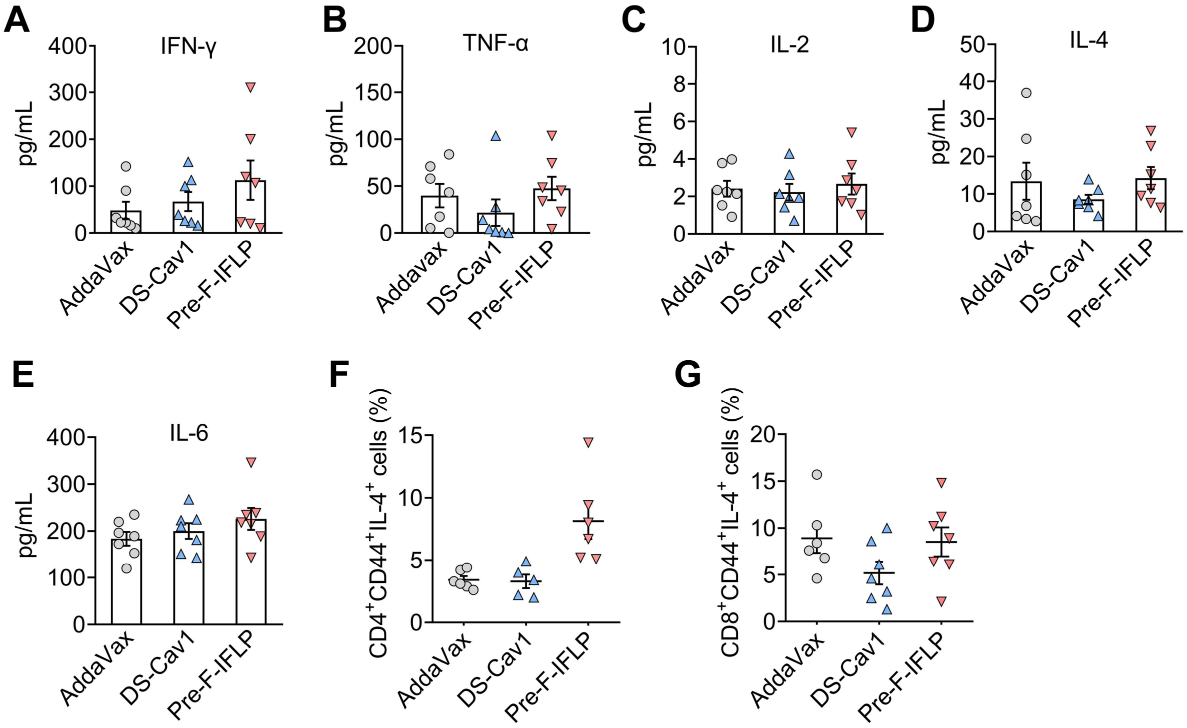


**Supplementary Fig. 2. Detection of immunoregulatory cytokines and lymphocyte immunophenotype after prime immunization.** Sera and spleens of mice were collected on day 7 after prime immunization. (**A**–**E**) Enzyme-linked immunosorbent assay (ELISA) was performed to detect the levels of IFN-γ, TNF-α, IL-2, IL-4, and IL-6 in the sera. Data are presented as mean ± SEM, n = 7 per group. (**F**, **G**) The spleens of immunized mice were harvested on day 7, and the lymphocyte immunophenotypes of the splenocytes were analyzed using flow cytometry. Data are presented as mean ± SEM, n = 6–7 per group.

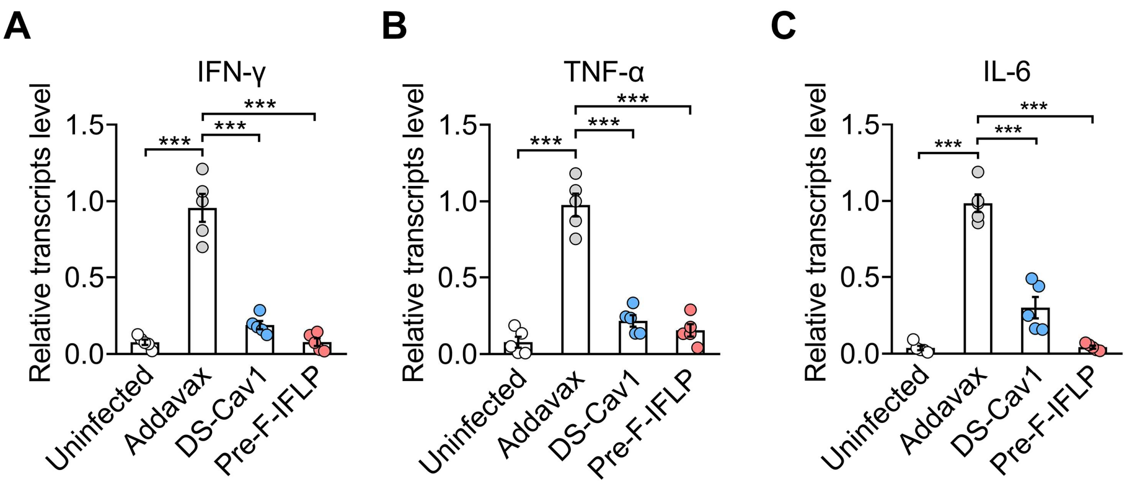


**Supplementary Fig. 3. Protective effects of pre-F-IFLP and DS-Cav1 immunizations against RSV-induced inflammatory responses.** Following three rounds of immunizations, the mice were challenged with RSV on day 55. Lung tissues were harvested and homogenized 5 days after infection. The levels of inflammation-related cytokines in the lung homogenates were determined by RT-PCR. (**A**–**C**) Each dot represents an individual mouse. Data are presented as mean ± SEM. Statistical differences between groups were analyzed using an unpaired two-tailed *t*-test. ***, $P < 0.001$.

**Supplementary Table 1. Mutation sites of RSV F variants**

| Variants | Mutation residues | CP/TM | Termination point | External trimerization motif |
|---|---|---|---|---|
| $Fwt_{ct}$ | None | With | 574 | None |
| F(ecto) | None | Without | 529 | None |
| △FP$_{ct}$ | Deletion of residues FLGFLLGVGS in fusion peptide | With | 574 | None |
| $DS_{ct}$ | S155C, S290C | With | 574 | None |
| $Cav1_{ct}$ | S190F, V207L | With | 574 | None |
| DS-$Cav1_{ct}$ | S155C, S290C, S190F, V207L | With | 574 | None |
| $DM_{ct}$ | N67I, S215P | With | 574 | None |
| $TM_{ct}$ | N67I, S215P, E487Q | With | 574 | None |
| SC-$DM_{ct}$ | N67I, S215P, GSGSG linker | With | 574 | None |
| SC-$TM_{ct}$ | N67I, S215P, E487Q, GSGSG linker | With | 574 | None |
| Pre-F-$IFLP_{ct}$ | N67I, S190F, V207L, S215P | With | 574 | None |
| Pre-F-$IFLPQ_{ct}$ | N67I, S190F, V207L, S215P, E487Q | With | 574 | None |
| △FP | Deletion of residues FLGFLLGVGS in fusion peptide | Without | 513 | T4 fibritin/Throm/6his/Streptag ll |
| Fwt | None | Without | 513 | T4 fibritin/Throm/6his/Streptag ll |
| DM | N67I, S215P | Without | 513 | T4 fibritin/Throm/6his/Streptag ll |
| TM | N67I, S215P, E487Q | Without | 513 | T4 fibritin/Throm/6his/Streptag ll |
| Cav1 | S190F, V207L | Without | 513 | T4 fibritin/Throm/6his/Streptag ll |
| DS-Cav1 | S155C, S290C, S190F, V207L | Without | 513 | T4 fibritin/Throm/6his/Streptag ll |
| Pre-F-IFLP | N67I, S190F, V207L, S215P | Without | 513 | T4 fibritin/Throm/6his/Streptag ll |
| Pre-F-IFLPQ | N67I, S190F, V207L, S215P, E487Q | Without | 513 | T4 fibritin/Throm/6his/Streptag ll |
